# Whi7/Srl3 polymorphisms reveal its role in cell size and quiescence.

**DOI:** 10.17912/micropub.biology.000661

**Published:** 2022-11-01

**Authors:** Shawna Miles, Linda L Breeden

**Affiliations:** 1 Fred Hutchinson Cancer Center

## Abstract

Whi5 and Srl3/Whi7 are related proteins that resulted from the whole genome duplication of *S. cerevisiae* (Wolfe and Shields 1997). Whi5 plays an Rb-like function in binding and inhibiting the late G1 transcription that promotes progression from G1 to S (Costanzo *et al.* 2004; de Bruin *et al.* 2004). Whi7 can also associate with G1 transcription complexes and promotes G1 arrest when overproduced (Gomar-Alba *et al.* 2017), but its transcription is primarily induced by stress (Ragni *et al.* 2011; Mendez *et al.* 2020). We have used polymorphisms in two laboratory yeast strains to uncover novel functions of Whi7 in log and quiescent cells. These include small cell size during log phase and defects in entry, maintenance and recovery from quiescence.

**Figure 1. Whi7 affects cell size and the production, longevity and recovery of quiescent cells.**
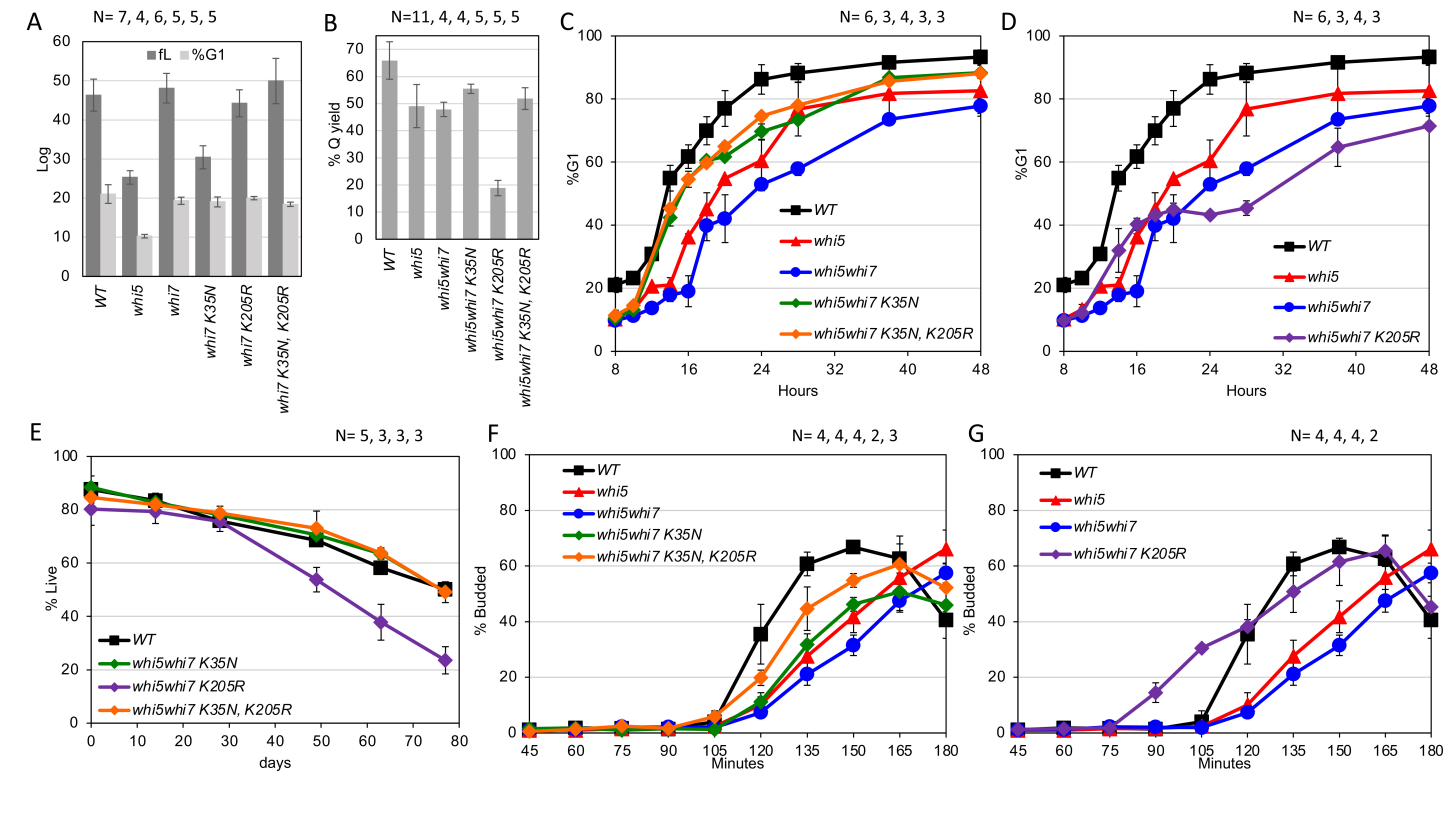
The genotypes are indicated in each plot and the number of replicates is listed above in the order listed in the plot. These strains have been assayed for (A) Cell size and percent of cells in G1 during logarithmic growth, (B) yield of quiescent cells purified after 7 days of growth, (C and D) percent of cells in G1 as cells transition from log phase (8 hrs) to stationary phase (48 hrs). (E) reports the percent of viable quiescent cells, purified from 7 day cultures and incubated with aeration in water for up to 77 days. (F and G) Purified quiescent cells resuspended in rich medium and monitored for budding over 3 hours.

## Description

Whi7 was originally identified as a high copy suppressor of Rad53 (Srl3), the DNA damage and replication stress checkpoint kinase (Desany *et al.* 1998). It has homology to Whi5, including all its cyclin-dependent kinase phosphorylation sites, which target Whi5 for inactivation, and its GTB domain, which enables Whi5 to bind and inhibit the G1 transcription complex of Swi4 and Swi6 (Travesa *et al.* 2013). There is considerable evidence that Whi7 plays a similar but lesser role to Whi5 in the inhibition of the G1 transcription and promoting G1 arrest (Gomar-Alba *et al.* 2017). Recent work suggests that the primary role of Whi7 is to promote G1 arrest in response to stress (Mendez *et al.* 2020). Cell wall and DNA damage, heat, and nutrient limitation induce Whi7 transcription through the C ell W all I ntegrity Pathway (Pkc/Slt2/Rlm1) (Quilis *et al.* 2021). This CWI pathway is required for the formation, longevity and recovery of quiescent cells (Miles *et al.* 2019). We have noted differences in the response to *WHI5* deletion in two lab strains S288c and W303. This led us to wonder if the Whi7 proteins in the two strains, which differ at two positions, could be responsible for these differences. We also wondered if Whi7 might play an important role in quiescence induced by nutrient limitation. To test this, we inserted each of the S288c substitutions into the Whi7 locus of W303. These substitutions are referred to as *whi7K35N* and *whi7K205R* , reflecting substitution of the lysines (K) in W303 with the asparagine (N) or arginine (R) found at that position in S288c.

In a W303 *WHI5* background, these Whi7 substitutions have little impact. They respond to nutrient limitation with G1 arrest just like wild type cells. Yield, longevity and recovery of quiescent cells is also wild type. However, *WHI5 whi7K35N* produces cells that are significantly smaller than wild type (p=.0009) during logarithmic growth (Figure 1A). *whi5* produces cells of a similar size, but in the case of *whi5* , this small cell size is correlated with a shorter G1 than wild type cells, as measured by percent of cells in G1 (p=.0001, Figure 1A). *whi7K35N* maintains a wild type percent of G1 cells, so failure to remain in G1 cannot explain its small size. This phenotype is not observed in the *whi7* null ((Jorgensen *et al.* 2002) and Figure 1A), nor is it conferred by *whi7K205R.* *whi7K35N, K205R* is also wild type size, so the K205R substitution rescues this K35N size phenotype.

Additional phenotypes can be observed in the absence of Whi5. As cells grow from log phase to stationary phase (Figure 1C and D), G1 arrest is delayed and incomplete in *whi5* and *whi5whi7* . This is in part due to the small size of these cells, which also delays glucose limitation from the medium (Miles *et al.* 2021). The *whi5whi7K35N* and the *whi5whi7K35N, K205R* mutants display an arrest profile more like wild type, suggesting that these alleles are at least as active as the W303 allele of *WHI7.* In contrast, *whi5whi7K205R* is more defective in G1 arrest than either *whi5WHI7* or the *whi5whi7* null late in the transition to stationary phase (p=.006 and .0007, respectively, at 28 hrs, Figure 1D.) In particular, *whi5whi7K205R* shifts from 10% to 40% of the population in G1 after 16 hours of growth, but then remains at 40% for the next 12 hours. *whi5WHI7* reaches 80% and *whi5whi7* reaches 60% G1 cells at this time. These data suggest that *whi7K205R* is exerting an antagonistic effect on Whi7 function after the diauxic shift when the glucose is exhausted from the medium. This negative influence is also neutralized by addition of the K35N substitution in *WHI7,* which also reaches 80% G1 cells (Figure 1C).

Consistent with the failure to arrest in G1, *whi5whi7K205R* produces about one third as many quiescent cells as the *whi5whi7* null (p=10 ^-6^ ), and yield is restored in the *whi5whi7K35N, K205R* mutant (p=0.13, Figure 1B). The quiescent cells that are produced by *whi5whi7K205R* have a shorter life span than wild type (p=.00003 at 77 days), and this phenotype is also rescued by *whi5whi7K35N, K205R* (Figure 1E) *.* Upon re-feeding, quiescent W303 cells show a 105 minute delay before they initiate budding. Deletion of *whi5* and *whi7* delays completion of this recovery cell cycle ((Miles *et al.* 2016) and Figure 1F.) Budding is initiated at about the same time as wild type, but completion of the process is delayed at least 30 minutes. In contrast, *whi5whi7K205R* accelerates cell cycle re-entry. It initiates budding 30 minutes before wild type (p=.003 at 105 minutes), and completes the process 15 minutes later. Based on previous work with log phase cells (Vallen and Cross 1995; Sidorova and Breeden 2002), it is likely that this accelerated cell cycle re-entry is evoking a sub-optimal S phase and results in a checkpoint-dependent delay in S or G2/M. *whi5whi7K35N, K205R* does not accelerate budding, indicating that the addition of K35N also neutralizes this phenotype.

We have uncovered some novel roles for Whi7 in log phase and in the transition into and out of quiescence. Unlike most studies which utilize null and overproduction alleles, we have introduced two naturally occurring *WHI7* polymorphisms found in a “wild type” lab strain S288c into a second “wild type” lab strain W303 and observed a surprising complexity of phenotypes. These mutations change the function and/or regulation of Whi7 in W303 and confer novel phenotypes. *whi7K35N* reduces cell size during logarithmic growth without affecting the length of G1. *whi7K205R* is defective in the production and longevity of quiescent cells and causes their premature cell cycle re-entry upon re-feeding. Both mutations display unique phenotypes that are largely ameliorated by addition of the second mutation, suggesting that evolution has been in play to moderate the impact of the single mutants.

Whi7 is associated with the ER and is also present in the nucleus from telophase to late G1 (Yahya *et al.* 2014). It associates with late G1 transcription complexes and can cause G1 arrest when overproduced (Gomar-Alba *et al.* 2017). It also interacts with Cdc28 and binds G1 cyclin promoters during replication stress (Travesa *et al.* 2013; Zhang *et al.* 2017). It is phosphorylated by Cdc28 and exported from the nucleus after start. Whi7 is an unstable protein, and its degradation is also cell cycle regulated and directed by ubiquitination through the SCF ^Grr1^ proteosome pathway (Gomar-Alba *et al.* 2017). Positions 35 and 205 in the Whi7 protein have not been implicated in any of these associations, but K35 is adjacent to a known phosphorylation site (Saccharomyces Genome Database). Both substitutions eliminate lysines and could prevent critical modifications (e.g. ubiquitylation, acetylation). Determining how these polymorphisms influence Whi7 expression, localization, interaction and modification, and how they neutralize the effects of each other, will provide new insight into Whi7 function and help define the Whi7 domains required for that function.

## Methods

*Yeast Strains and Growth Conditions*

All strains are isogenic with BY6500, the prototrophic version of W303 (Li *et al.* 2009). *WHI5* and *WHI7* were deleted using pFA6a- *HIS3MX6* and pFA6a- *KanMX* , respectively (Longtine *et al.* 1998). The S288C allele of *WHI7* was cloned into pRS306 (pBD3088) then integrated via transplacement (Scherer and Davis 1979) into a *ura3-1* version of BY6500. The *URA3* was healed by homologous gene replacement. Growth curve and stationary phase cultures were grown as described in (Miles *et al.* 2016).

*Cell Processing*

Quiescent cells were harvested as described in (Miles *et al.* 2016). Flow cytometry was processed as stated in (Li et al., 2013). The percent G1 was calculated as described in (Miles *et al.* 2016). Cell size was measured with a Z2 Beckman Coulter Counter (Beckman Coulter, Brea, CA). Size reported in femtoliters (fL) reflect the modal cell size. The longevity and viability assays of quiescent cells were performed as described in (Li *et al.* 2009). For quiescent cell cycle re-entry, the process was detailed in (Li *et al.* 2013).

## Reagents

BY6500 *MATa can1-100 rad5 ssd1-d*

BY7326 *MATa can1-100 rad5 ssd1-d whi5Δ::HIS3*

BY7332 *MATa can1-100 rad5 ssd1-d whi7Δ::KanMX*

BY7334 *MATa can1-100 rad5 ssd1-d whi5Δ::HIS3 whi7Δ::KanMX*

BY7347 *MATa can1-100 rad5 ssd1-d whi7 K35N*

BY7343 *MATa can1-100 rad5 ssd1-d whi7 K205R*

BY7345 *MATa can1-100 rad5 ssd1-d whi7 K35N, K205R*

BY7338 *MATa can1-100 rad5 ssd1-d whi5Δ::HIS3 whi7 K35N*

BY7349 *MATa can1-100 rad5 ssd1-d whi5Δ::HIS3 whi7 K205R*

BY7351 *MATa can1-100 rad5 ssd1-d whi5Δ::HIS3 whi7 K35N, K205R*
